# Intelligence Is beyond Learning: A Context-Aware Artificial Intelligent System for Video Understanding

**DOI:** 10.1155/2020/8813089

**Published:** 2020-12-23

**Authors:** Ahmed Ghozia, Gamal Attiya, Emad Adly, Nawal El-Fishawy

**Affiliations:** Computer Science and Engineering Department, Faculty of Electronic Engineering, Menoufia University, Shibin El Kom, Menofia Governorate, Egypt

## Abstract

Understanding video files is a challenging task. While the current video understanding techniques rely on deep learning, the obtained results suffer from a lack of real trustful meaning. Deep learning recognizes patterns from big data, leading to deep feature abstraction, not deep understanding. Deep learning tries to understand multimedia production by analyzing its content. We cannot understand the semantics of a multimedia file by analyzing its content only. Events occurring in a scene earn their meanings from the context containing them. A screaming kid could be scared of a threat or surprised by a lovely gift or just playing in the backyard. Artificial intelligence is a heterogeneous process that goes beyond learning. In this article, we discuss the heterogeneity of AI as a process that includes innate knowledge, approximations, and context awareness. We present a context-aware video understanding technique that makes the machine intelligent enough to understand the message behind the video stream. The main purpose is to understand the video stream by extracting real meaningful concepts, emotions, temporal data, and spatial data from the video context. The diffusion of heterogeneous data patterns from the video context leads to accurate decision-making about the video message and outperforms systems that rely on deep learning. Objective and subjective comparisons prove the accuracy of the concepts extracted by the proposed context-aware technique in comparison with the current deep learning video understanding techniques. Both systems are compared in terms of retrieval time, computing time, data size consumption, and complexity analysis. Comparisons show a significant efficient resource usage of the proposed context-aware system, which makes it a suitable solution for real-time scenarios. Moreover, we discuss the pros and cons of deep learning architectures.

## 1. Introduction

Current smartphones come with great hardware and software capabilities. These devices gave their owners the ability to become active online publishers. Smartphone owners are media producers through their YouTube channels, authors, journalists on their personal Facebook profiles, and news reporters via their tweets on Twitter. All these productions come in the form of multimedia content. Almost every YouTube video comes as an audio and visual signal synchronized with a subtitle, released through a YouTube channel, and may be within a playlist as well. The audience may watch YouTube and express their interpretation of the story told via comments, likes, and dislikes. The same scenario occurs in other social media platforms such as tweets on Twitter, posts on Facebook, and photos on Instagram.

Generally, multimedia production and consumption are instantaneous. YouTube daily users count to more than two billion, with one billion hours watched daily [1]. This heterogeneous multimedia production comes with a contextual container of time, emotions, geographical location, and events before and after the media file.

Every media producer is a human being with a story to tell. While that story is composed, the author uses every aspect of his human intelligence. The story consumer is also a human who will use all his intelligence toolbox to understand the concept behind the story told. Human intelligence is a multidimensional toolbox. It includes common sense, innate knowledge, approximations, learning capability, context-awareness, and reasoning. The problem now is how to make the machine intelligent enough to understand the video stream.

Recently, researchers tend to use deep learning to solve such problems. However, it is neither fair nor possible to expect a human-like understanding from machines based only on deep learning architectures. So, rich artificial cognitive models are needed for a deep understanding of the concepts behind media productions, charging them with every possible aspect of human intelligence. That is the path toward real artificial general intelligence which could exist in our daily life and bring real value.

**Figure 1:**
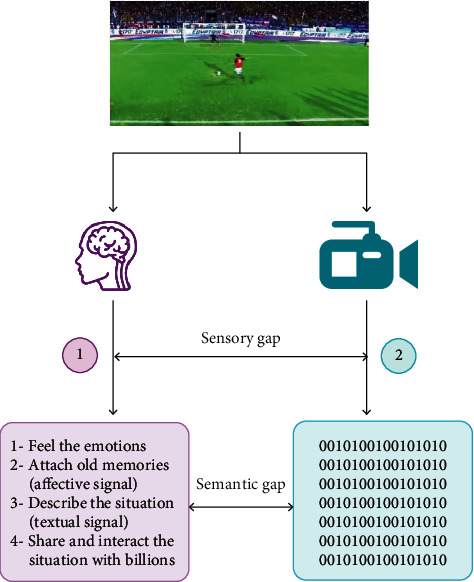
Semantic gap between human and computer perception of the physical world. (1) Human perception is represented by high-level features (concepts): watch the penalty (visual signal, scream (audio signal), and talk with the crowd (natural language processing). (2) Machine perception is represented by low-level features (texture, color, resolution, and encoding).

To make this possible, we need to mind the semantic gap [2], shown in Figure 1, between the low-level features that represent the audio, visual, and textual content of the video and the high-level concepts as perceived by human cognition.

**Figure 2:**
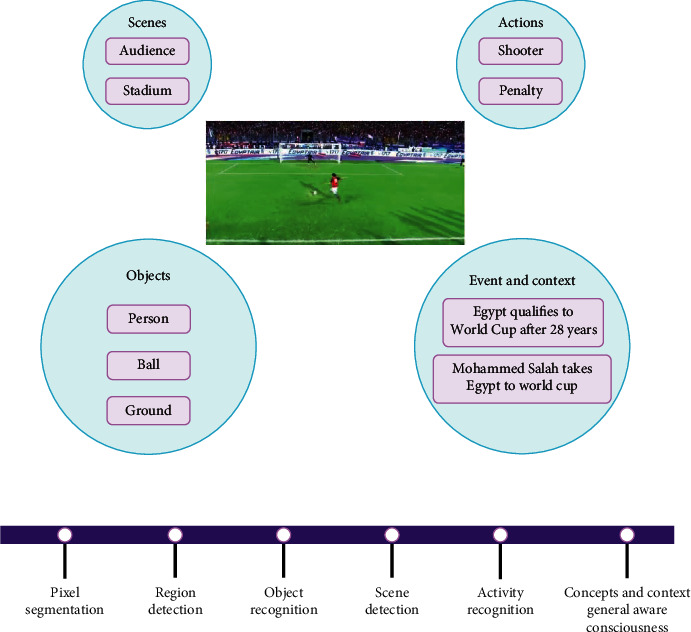
Video understanding core problems.

Figure 2 shows a goal scored by a player on his official Twitter account. A supporter will celebrate that goal while watching the match (visual signal), listening to the commentator (audio and sound signals), and reading comments (textual data). To make such a video file available and reachable to the concerned target audience, a human-like cognition architecture is needed to process all the signals of the video file, correlate them to the surrounding context, and recognize the different actions within the scene. The figure summarizes video understanding core problems as video representation (pixel segmentation), region detection, action recognition, and storytelling.

This article presents a transition phase between understanding of video content era and our proposed framework on video context-aware understanding. The proposed context-aware cognition system utilizes metadata, emotions, comments, title, description, temporal data, spatial data, and likes/dislikes playing a vital role in the interpretation of concepts within the video. The proposed system consumes less time, computing power, and data compared to the classical learning approach. The main contributions of this paper can be summarized as follows:

1. In the context of video understanding, we define an artificially intelligent machine as a machine capable of deciding the true meaning of the video file. We describe artificial general intelligence (AGI) as a heterogeneous process that includes not only learning but also innate knowledge, approximation, and context-awareness.
2. Spot the light on the limitations of deep learning, not only its capabilities.
3. Design and implement a context-aware system for video understanding. The proposed system diffuses the video context into weighted concepts, emotions, categories, and temporal information about the video.
4. Design a benchmark of real videos (RealVids). RealVids represents real-life videos we watch daily on social media platforms. RealVids is not a fine-tuned dataset that acts properly only with well-trained algorithms.
5. The proposed context-aware system and classical deep learning technique are evaluated against the RealVids benchmark. Performance analysis is carried out via objective and subjective comparison of recognized concepts. The context-aware system is compared with the deep learning baseline in terms of data retrieval time, time consumed to generate concepts, data size consumed to generate such concepts, and complexity analysis of both algorithms.

The remainder of the paper is organized as follows. In Section 2, we focus on the accomplishments and limitations of deep learning architectures in video understanding. Then Section 3 introduces the definition of artificial intelligence as a decision-making process, heterogeneity of artificial general intelligence, and context-awareness. We introduce the main body and implementation of the context-aware video understanding in Section 4. Next, we demonstrate the experimental results and give some discussions and evaluation metrics of the results in Section 5.  Section 6 summarizes our conclusions and presents research extensions for the proposed work.

## 2. Related Work

The objective of this section is to discuss the core problems of video understanding, accomplishments, and limitations of deep learning architectures toward these problems.

**Figure 3:**
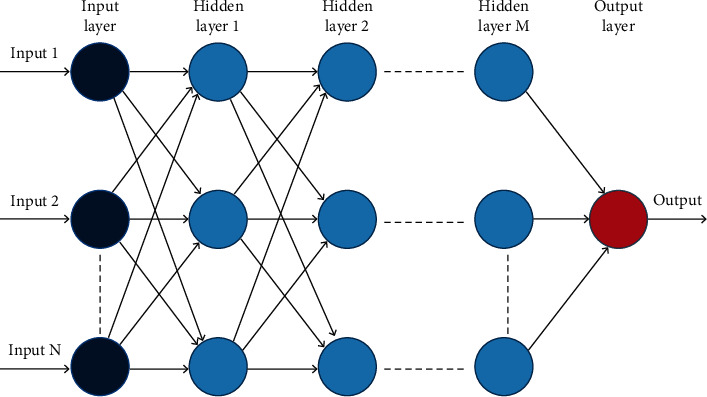
General architecture of deep learning.

Deep learning models are systems of multiple neural layers that build by improving the level of abstraction starting from the first layer and then onto the next layer, as shown in Figure 3. These strategies enhance the cutting-edge research in video understanding including audio, text, and visual signals [3]. Deep learning achieves noticeable progress in the fields of pattern recognition [4, 5], beating humans at games level [6], neuroradiology [7], healthcare [8], FEA design and misfit minimization [9], travel decision frameworks [10], data-driven Earth system science [11], and analysis of graph signals [12].

### 2.1. Video Representation: Segmenting Pixels and Region Detection

In 2011 [13], videos were represented using handcrafted features. Handcrafted features suffered from camera motion and illumination change in video, contained no high-level semantic information or high dimensionality, and were too expensive for real-time computation. By 2014, 2D Convolutional Neural Networks (CNNs) were introduced. The authors in [14] processed video files as a bag of short fixed-size clips, thus extending the network connections in the time domain. They explored approaches for fusing information over the temporal dimension through the network. In 2015, [15] introduced long-term recurrent convolutional networks, where the outputs of a 2D CNN are fed into a stack of Long Short-Term Memory (LSTM) networks. It neglected low-level motion information while being efficient in tasks related to activity recognition and video description. Some research trials went for 3D CNNS [15] but it turned out to be a computationally expensive and time-consuming task. In comparison, fine-tuning 2D CNNs was more beneficial than training a 3D CNN.

### 2.2. Action Recognition

Understanding human activities in visual information is based on progress in other research areas such as object recognition, semantic segmentation [16], and domain adaptation. Action recognition in videos had advanced from tailored solutions for specific problems to general-purpose solutions that can learn from millions of videos and apply to daily activities. Given the broad scope of applications from video surveillance to logo detection, many scientific contributions were achieved. Action recognition covers a broad scope of crucial daily life events including web video search, video surveillance, medical diagnosis, elderly care, and sports analytics. Oxford dictionary defines an action as “the fact or process of doing something, typically to achieve an aim” and activity as “a thing that a person or group does or has done.” Deep learning models had been developed for action recognition in video. These models could fall into three categories.

#### 2.2.1. Spatiotemporal Networks

The convolutional design adequately uses the picture structure in diminishing the search space of the network by pooling and weight-sharing. Pooling and weight-sharing also help bring robustness to the scale and spatial dimensions. Breaking down channels learned by CNN designs tells that the first layers learn low-level features (such as SIFT features), while top layers learn high-level semantics [17], making CNNs generic feature extractors. Reference [18] suggests handling the problem of action recognition through a cascade of convolutional networks and then a recurrent neural network (RNN) known as Long Short-Term Memory (LSTM). To classify activities, the authors in [19] propose inputting the LSTM network with features computed from a 3D convolutional network. The two networks, that is, 3D convolutional network and the LSTM networks, are prepared independently. That is, first the 3D convolutional network is prepared to utilize annotated activity information. Once the 3D convolutional network is acquired, the convolutional features are utilized for training the LSTM network.

#### 2.2.2. Multiple Stream Networks

In visual perception, the ventral stream of the human visual cortex processes objects properties, for example, appearance, shading, and personality. The movement of an object and its location are taken care of independently through the dorsal stream [19]. A class of deep neural systems are formulated to isolate appearance-based data from movement-related ones for activity acknowledgment [20]. Two parallel systems are utilized for activity recognition. The alleged spatial stream organizes crude video frames, while the temporal stream network gets optical stream fields as information.

Different research challenges, such as the ActivityNet Large-Scale Activity Recognition Challenge, are prepared by researchers to let algorithms recognize actions in videos [21, 22]. The main challenge in such datasets aims to recognize daily life and high-level semantics from user-generated videos as those found on web portals [22]. Algorithms would compete to achieve accurate predictions of actions in videos. Almost all methods in these competitions use deep learning architectures, such as Action Pyramid Networks [23], Deep Bag of Frames, and recurrent neural networks model families [24] or Large Ensembles of Heterogeneous Neural Networks [4].

### 2.3. Limitations of Deep Learning Architectures

Systems that depend on deep learning have to generalize over the training data they have seen. For deep learning models to generalize well, they need to train over large amounts of data and the test data must be similar to the training data. This sort of learning works well at finite worlds such as already organized datasets where training and testing datasets are precisely chosen. However, recognizing concepts in a video file requires a human-like intelligence that can generalize abstractions from raw and incomplete data. Trying to achieve generalization and abstractions, deep learning architectures had the following limitations:

1. Currently, the largest artificial neural networks, built on supercomputers, have the size of a frog brain (about 16 million neurons). On the other hand, the human brain contains 100 billion neurons, passing signals to each other via as many as 1,000 trillion synaptic connections [25].
2. Deep learning architectures learn from videos and images captured by cameras with 45 megapixels, at their best, while the human eye has a resolution of 576 megapixels [26].
3. Deep learning is data-hungry and works best when there are millions and billions of training examples [27], while humans are much more efficient in learning abstractions and rules than deep learning [28].
4. Deep learning suffers from a learning rate that stops at a certain limit of data size [29].
5. Deep learning cannot represent hierarchical structures where large structures are constructed from smaller ones [30], such as natural language statements and actions occurring in a video file.
6. Deep learning represents a black box for researchers and system designers. The millions of parameters within the neural network are not known in an interpretable human way; all we know is their geography within the network (*i*^*th*^ node in layer *j*) in network module *L*. The importance of deep learning transparency depends on whether the deep learning models are self-contained standalone systems [31] or they need to fit in the context of larger systems. The need for an explanation is crucial when the decision is used in a critical context such as military, finance, and health. A score will not be enough. A detailed explanation of the theories behind this score would be necessary.
7. The task of classifying a consumer as creditworthy justifies the importance of the explainable decision. No one, neither credit applicant nor banker, should be satisfied with a system that does not explain its conclusions. A raw score that represents the decision will not be enough justification. Both the customer and the banking system will need an explanation for such a score, the training data, and the motives behind it. The same comments apply to those making the decisions. In arenas with more earthshaking implications such as international relations, this should be part of the decision-making best practices.
8. Deep learning does not integrate directly with prior knowledge because it depends on the blind correlation between features rather than abstractions [32]. Deep learning is working well with packaged problems where training and testing examples are already organized into folders. Real life is not organized that way and humans do not get their learning arranged into folders.
9. Deep learning learns correlations between input and output features but with no inherent representation of causality [33]. Perhaps deep learning is not targeted toward those problems.
10. Deep learning fits well in stable organized worlds such as the board game Go [6] which have unvarying rules. Deep learning does not fit well in constantly changing worlds such as economics, politics, or movies [34].

## 3. Background

In our method, we rely on expanding the video understanding process from learning to context-awareness. We handle video understanding as a decision-making process where we want to decide which concepts represent the video message. Therefore, we provide the necessary background on the definition of artificial intelligence as a decision-making process, the heterogeneity of artificial general intelligence (AGI), and context-awareness.

### 3.1. AI: From Deep Learning to Artificial General Intelligence

It is interesting to see where the previously mentioned deep learning techniques originally come from. Rosenblatt introduced the concept of the one-layer architecture of the perceptron learning algorithm [35] and Sutton introduced the concept of reinforcement learning [36], both at Psychological Review. Hinton introduced Boltzmann machines [37] and Elman introduced the first simpler version of LSTMs [38], both at Cognitive Systems. Hinton introduced the backpropagation algorithm for training multilayered neural networks [39] at Nature. We see papers published in the fifties and eighties in psychology and cognition journals. That is, cognitive science and psychology formalized fundamental insights about how humans might learn that led to all the deep learning architectures we are witnessing now. Our vision for the future of artificial intelligence initiates its seeds from looking deeper into these two fields and comes up with potential approaches toward artificial general intelligence.

#### 3.1.1. Definition of AI

Artificial intelligence has got different definitions from different perspectives [40]. We chose to define intelligence following the psychological perspective [41]: as the ability of autonomous decision-making without external intervention. A robot detecting a glass to move it from one place to another is making a decision: a decision whether it is the glass or not or a decision whether the identified place is the correct one or not. When a query is sent to the Google Web search engine, Google responds with an ordered list of results. Putting those results in that order is a decision-making process as well. We can reason in the face of incomplete and imprecise information.

#### 3.1.2. Heterogeneity of AGI

Human intelligence is multidimensional. It includes emotional intelligence, linguistic intelligence, social intelligence, common sense, context-awareness intelligence, perception, and approximation. The AGI needs a general cognitive model, not a statistical approximation model, because it is not possible to understand the world within a probability distribution model. Thus, methods different from deep learning are needed, methods that not only need fewer data to learn but also are able to represent abstract knowledge.

*Innate knowledge* is essential knowledge that typical individuals are anticipated to have regardless of whether they do not have the foggiest idea of what precisely it is. Innate knowledge is the mystery of how to settle on choices about novel cases for which there are not many or no examples to learn from. There is sufficient proof, both social and mental, that biological creatures start from rich starting points, even before learning starts [42]. Starting points that are rich containers of objects, actions, and space result in a better learning experience. The richer your start point, the richer you learn. English philosopher John Locke was wrong; we are not blank slates [43]. Researchers of machine learning and deep learning tend to improve AI by improving the learning algorithms and ignore innate knowledge. The usual presumption is that we need to fix the learning paradigm, not to adapt to new innate machinery, knowledge, and representations.

*Approximations* had been of interest to AI from the early days. For instance, Edward Feigenbaum wrote the following [44]: “A useful rule of thumb used by human beings in most of their problem-solving is this: attack a new problem by methods that have solved similar problems in the past. The criteria for “similarity” may themselves be heuristic.” Humans use approximation daily for perception, reasoning, cognition, and making decisions. Humans do not memorize everything about the people they know, the cities they travel to, or the daily life methods they adopt. The list of hardwired cognition is limited. Besides that, the ever-changing messy world makes approximation the pragmatic way for humans to interact with the world.

### 3.2. Context-Awareness

The word “context” has Latin roots, where the word “con” means to join together and “texere” means to make or to weave, implying weaving together the circumstances that form the setting of a scenario. The context of concern could be user, computation, time, or cognitive. Context-awareness is the ability of a system to give user-relevant information by utilizing the contextual information of the concerned event. Context is a multidimensional feature space that evolves with time. Considering the context of a situation into consideration brings insights and intuitions that could help make better decisions and understanding. The context supports decision-making by filling the gaps in uncertain environments. Context means different things to different people; one well-cited and accepted definition is the following: “Context: any information that can be used to characterize the situation of entities (i.e., whether a person, place, or object) that are considered relevant to the interaction between a user and an application, including the user and the application themselves. Context is typically the location, identity, and state of people, groups, and computational and physical objects” [45]. Context-awareness is developed through three stages:

1. Context representation: it defines how to represent the elements of context and how to define them.
2. Context determination: it defines how to determine the context elements in an uncertain environment.
3. Context analytics: it defines how to summarize and predict the context in an agile way.

## 4. A Proposed Context-Aware System for Video Understanding

In this section, we present an implementation of a context-aware understanding of the video file. We compare context-aware video understanding with deep learning video recognition. The aim is to understand the concepts in a typical social media video file that we see daily. We choose a set of videos that represent a topic from YouTube, in this case “screaming kid.” YouTube is an ideal representation of social media platforms. It allows publishers to publish videos with titles, descriptions, and metatags. YouTube audience can comment on, like, and dislike the video, thus developing a context around the video. The same environment exists for Facebook posts, Twitter tweets, and Instagram photos. The main feature that distinguishes YouTube is its publicity as all videos are public and available to everyone, which is not the case for content on other social media platforms.

**Figure 4:**
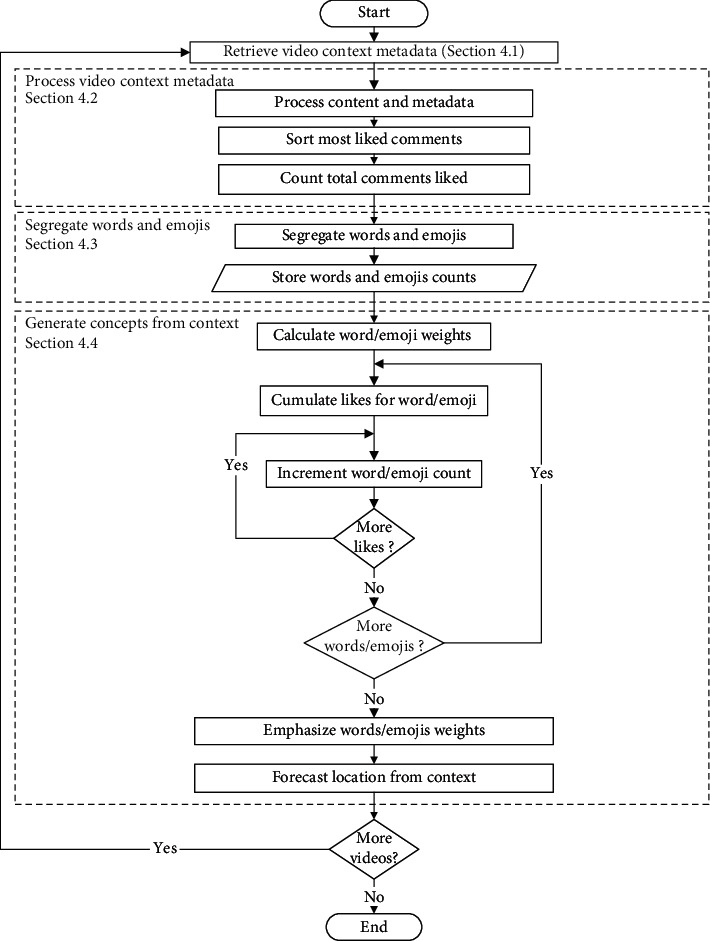
Context-aware video understanding.

Figure 4 shows how the proposed context-aware system flow is working. The system consists of these main stages:

1. Retrieve video context metadata
2. Process video context metadata
3. Segregate words and emojis
4. Generate concepts from context

The start is with retrieving video content and metadata from an external source, YouTube in this case, and the end is with extracting concepts from the video's context.

Video context is built by harvesting and inspecting the metadata available for YouTube videos through the YouTube Data API [46]. The algorithm extracts the title, description, and the top relevant 100 comments for each video sorted by the most relevant. Comment relevancy is based upon counting likes. The whole algorithm is developed using Python. In the following subsections, we will describe the details of each step.

### 4.1. Retrieve Video Context Metadata

The system starts by fetching video information from an external source, YouTube in this case.  Algorithm 1 is used to retrieve YouTube content and metadata using YouTube Data API [46] with Python and pafy [47] (Python library for retrieving YouTube content).

### 4.2. Process Video Context Metadata

Comments are ordered in a descending order. The video title, top 100 comments, and description are filtered by removing stop words. After filtering, the system uses NLTK (Natural Language Processing Toolkit) [48] functions to count word occurrences in the context.  Algorithm 2 is applied to calculate such occurrences.

### 4.3. Segregate Words and Emojis

Segregating words and emojis from the list enables detecting the global social-emotional expression within the video context.  Algorithm 3 segregates emotions hiding into the extracted concepts using emoji (emoji extraction package) [49]. Emojis are generated and used for forecasting the global social-emotional state within the context.

### 4.4. Generate Concepts from Context

After creating lists of words and emojis that summarize the context, the weight for each word and emoji is calculated. The weight of each concept is estimated based on the ratio of the word occurrence to the total occurrences of all the words in the context. In this way, the weights are valued relative to the total counts of every extracted word and emoji.

We define a function

(1) $$\begin{array}{c} \text{Num\_Likes}:C⟶\mathbb{R}, \end{array}$$

where domain *C* is the set of all extracted comments.

(2) $$\begin{array}{c} n_{i}=\text{Num\_likes}\left(c_{i}\right)\;\forall i\left(1\leq i\leq \left|C\right|\right), \end{array}$$

where |*C*| is the cardinality of the finite set C. We construct a set of words *w*_*i*_ belonging to each comment *i*.

(3) $$\begin{array}{c} w_{i}=\left\{x:x\text{ is a word that belongs to comment }i\right\}. \end{array}$$

So, the set of all words *W* is

(4) $$\begin{array}{c} W=\overset{i=\left|C\right|}{\underset{i=1}{\cup }}w_{i}. \end{array}$$

Word frequency within the context is calculated according to equation (6), where *C* is the set of comments.

Algorithm 4 estimates word frequencies and the result is the concepts within the context. Extracted concepts are calculated as the intersection between keywords in video metadata and comments.

(5) $$\begin{array}{c} \text{Word\_frequency}:W⟶\mathbb{R}, \end{array}$$

(6) $$\begin{array}{c} \text{Word\_frequency}\left(x_{j}\right)=\sum_{i=1}^{i=\left|C\right|}I\left(i,j\right)*\text{Num\_Likes}\left(c_{i}\right), \end{array}$$

where the indicator variable *I*(*i*, *j*) is

(7) $$\begin{array}{c} I\left(i,j\right)=\left\{\begin{array}{cc} 1, & \text{if }\left(\;\right.x_{j}\in c_{i}\left.\;\right),\\ 0, & \text{if }\left(\;\right.x_{j}\notin c_{i}\left.\;\right). \end{array}\right. \end{array}$$

Equations (6) and (8) and (10) emphasize the concepts' weights. The more likes a word is given, the more chance for the word to be a key concept in the context. Equation (6) estimates the total occurrences for each word within the context. The total count of all the words in the context is the summation of each word occurrences as expressed in equation (8). Finally, the weight of each word within the context is calculated as the word frequency divided by total count of all words within the context as described by equation (10). The top 5 weights are selected as the representative concepts of the context.

(8) $$\begin{array}{c} \text{total}=\sum_{j=1}^{j=\left|W\right|}\text{Word\_frequency}\left(x_{j}\right), \end{array}$$

(9) $$\begin{array}{c} \text{Word\_Weight}:W⟶\mathbb{R}, \end{array}$$

(10) $$\begin{array}{c} \text{Word\_Weight}\left(x_{i}\right)=\frac{\text{Word\_frequency}\left(x_{i}\right)}{\text{total}}. \end{array}$$

## 5. Results and Discussion

### 5.1. Experimental Settings

#### 5.1.1. Experimental Benchmark: RealVids versus Datasets

The common practice of the research community is to evaluate algorithms and proposed theories against already prepared datasets such as YouTube-8M Large-Scale Video Understanding Challenge [21] and the ActivityNet Large-Scale Activity Recognition Challenge [22]. We consider the following critics toward this approach:

1. Learning from millions of videos is not a feasible solution to build AI systems that exist in daily life. Real-life situations that meet us every day are not frequently available in terms of millions or even hundreds of examples. To build real daily life AI machines, systems should need fewer examples.
2. Currently, existing video datasets require huge computing power [50] which makes it an unfeasible solution for all day-to-day AI applications. Achieving applicable and efficient video understanding shall adopt less needy systems.
3. Recent research claimed that research results from already prepared datasets are not reproducible [51]. Recommendations for more naturalism are increasing [52].

In response to the above critics, we designed a new test environment called RealVids, described in Table 1. RealVids is a collection of videos from YouTube. A query for the target topic is sent, in our case “screaming kid,” and ten videos are chosen from the top twenty results. These chosen videos vary in category, context, content, and duration to cover a wide range of topics.

#### 5.1.2. Environment

The proposed system is developed and tested on a Linux machine (Ubuntu 18.4). The hardware specifications are Intel Core I7-6500U @2.5 GHz, 8 GB RAM.

#### 5.1.3. Baseline

The “screaming kid” collection is trajected against the action recognition deep learning model of the Moments in time dataset [53]. According to the team behind it, “Moments is a research project in development by the MIT-IBM Watson AI Lab. The project is dedicated to building a very large-scale dataset to help AI systems recognize and understand actions and events in videos.” The dataset and its models represent the state-of-the-art performance of deep learning architectures for action recognition in videos. The dataset is over 1,000,000 labeled videos collected from ten different sources and trained over the ResNetI3D-50 model.

### 5.2. Results

Table 2 presents concepts extracted by the context-aware system on the RealVids dataset.  Table 3 presents other extracted metaconcepts such as emotions, spatiotemporal and, categorical information.

All videos are retrieved in response to the “screaming kid” query, but the content of the video message is different from one video to another. The context-aware system interpreted the video implied message from the context. For example, videos 2, 3, 4, and 6 represent a violent scene, but only 2, 3, and, 6 are actual violence. Video 4 represents an online video game. Concepts extracted from the context of 2, 3, and 6 represented such violence. These concepts included psycho, ruin, murder, scream, flight, annoy, and brat. Emotions of video 2 “joy and laughing” reflected the comedy behind the scene. Emotion of video 3 “Insane” reflected the real violence of the court scene. Meanwhile video 4 was represented by much fewer violent concepts such as gmod and rage. Emotions of video 4 “joy and laughing” reflected the video as just an online game.

Video 1 concepts extracted from the context came in accordance with the video message, such as a parent and learn. Extracted emotions such as thumbs up and proud express user satisfaction. Video 10 is a funny homemade video. Concepts extracted from the context such as funny, reaction, laughing, and joy expressed the real message behind the screaming kid in the video.

Videos 5 and 9 were modified advertisements with air rape and a song, accordingly. This mix-up caused disjoint discussions through the video comments. The context-aware system was unable to extract any meaningful concepts or emotions. Videos 7 and 8 did not contain any contextual information, so no concepts or emotions were extracted.

Categories of videos 2 and 4 integrated with extracted concepts giving a complete understanding of the video message. Categories and concepts of videos 1, 3, 6, and 10 came matching each other.

During the experiments, we realized that the context-aware system performs perfectly for videos that have rich metadata and objective discussions and comments that fulfill our defined criteria for the targeted media. On the other side, the context-aware system fails in extracting concepts from the context of poor metadata like a small number of comments or low levels of interaction, and this is visible for videos 7 and 8 in Table 2. For further trials to extract concepts from the context, experiments were extended to include less weighted concepts that have a low-level score but still valuable to consider it in the results. From these results, we see that the context-aware system is a perfect solution that helps in understanding metadata-rich contexts in a time record with the elimination of training time, also without requiring high hardware resources.

The context-aware system achieved these results efficiently in terms of retrieval time, processing time, consumed data size, and algorithmic complexity. The next section will discuss these metrics and their implications.

### 5.3. Discussion

The objective and subjective comparisons of the deep learning algorithm and the proposed technique are presented in Table 4.

The objective comparison shows how the proposed technique outperforms the classical learning one. Concepts extracted from the context were affirmative and more confirmed than those recognized by deep learning. This proves that the context-aware video understanding is more precise than the video understanding depending on the video content only.

The subjective point of view reveals a cutting-edge comparison between the generality of the classical learning technique and the definite clear and determined results of the proposed technique. The context-aware system recognized not only concepts but also emotions around the video, temporal, and categorical information.

Unfortunately, context-awareness was not able to predict any spatial information, though. Social media producers and users did not leave enough data about their geographical location, probably for privacy concerns.

Table 4 shows the results of the context evaluation. It can be seen that the actions recognized by the deep learning architecture are more general and broader on topic. They do not relate directly to the core message of the video. Concepts concluded from the context (title, description, and top comments) were more specific about the topic and tell what was really happening.

### 5.4. Evaluation Metrics

#### 5.4.1. Retrieving Time Comparison

**Figure 5:**
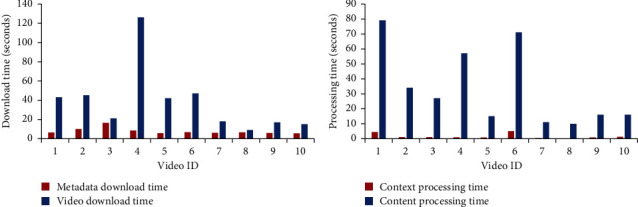
Evaluation metrics: retrieving time comparison and processing time comparison. (a) Time consumed for retrieving video context metadata versus video content, measured in seconds. (b) Processing time for video context metadata versus video content, measured in seconds.

Table 5 and Figure 5(a) show the time required for retrieving videos' context metadata and video content. These comparisons show the difference between average duration required for metadata download (7.812 seconds) and that required for content download (38.3 seconds), which saves 90% of time required for data retrieval.

#### 5.4.2. Processing Time Comparison

Table 6 and Figure 5(b) show the time required for processing videos context metadata and video content. These comparisons show the difference between average duration required for metadata processing (1.6 seconds) and that required for content processing (33.6 seconds), which saves 94% of the time required for data retrieval.

The context-aware system processes textual data to extract concepts, while the deep learning approach processes visual signal. The textual data describing a video is smaller than the visual signal for the same video. This explains the improved retrieval and processing time by the context-aware approach in comparison with the deep learning approach.

#### 5.4.3. Data Size Comparison

Comparing the results, the power of context-awareness analysis for real-time videos is noticeable. The context-aware system developed an understanding of the video message without trained models and without dataset dependencies.  Table 7 shows the size of processed data to generate concepts in both deep learning and context-awareness.

All results achieved by the context-aware system did not depend on trained models or big size datasets in contrast to the deep learning. Also, there was no training time for the context-awareness system.

#### 5.4.4. Complexity Analysis Comparison

For a machine-independent comparison, we carried out a complexity analysis between our proposed context-aware algorithm and the ResNet-50 architecture.

The overall complexity could be introduced as the complexity measure for all the four building blocks of applied algorithms.  Algorithm 1 fetches video information with O(1) complexity.  Algorithm 2 filters the metadata and the time complexity is O(n).  Algorithm 3 complexity is *O*(*n*^2^) to extract words and emojis. Algorithm 4 is calculating the total weights for extracted context actions and sorting them with the complexity of *O*(*n*^2^ + *n*). The overall complexity of the proposed system is described by equation (11) as result of *O*(1) + *O*(*n*) + *O*(*n*^2^) + *O*(*n*^2^ + *n*).

(11) $$\begin{array}{c} O\left(n^{2}\right). \end{array}$$

Hence, in this comparison, the total complexity of the proposed system is *O*(*n*^2^).

Equation (12) introduced in [47] shows that the complexity of the CNN architecture models depends on the depth of layers. Taking *l* as the convolutional layer index, *d* is the depth (number of convolutional layers), *n*_l_ is the number of filters (also known as “width”) in the *l*-th layer, *n*_l-1_ is the number of input channels of the *l*-th layer, *s*_l_ is the spatial size (length) of the filter, *m*_l_ is the spatial size of the output feature map, and this time complexity applies for both training and testing time.

(12) $$\begin{array}{c} O\left(\sum_{l=1}^{d}n_{l-1}*s_{l}^{2}*n_{l}*m_{l}^{2}\right). \end{array}$$

As a result, the complexity comparison had evolved to favor the context-aware system from an execution perspective due to the benefits of reducing the size of data to be processed, unnecessary training models, and the elimination of training time. Also, the context-aware system cares about human feedback and emotions around the video, which will lead to a better understanding of the video in a valid real context.

#### 5.4.5. Limitations

First, we observe that some videos may have no context metadata so extracting concepts become not possible.

Second, it becomes difficult to extract concepts from sparse context metadata. An example of a sparse context is when the social media audience may fill the video context with their emotions more than their thoughts. In this case, context-aware video understanding technique could be able only to extract emotional states rather than concepts.

## 6. Conclusions and Future Work

The presented research work introduces high-performance and precision context-aware video understanding technique. This context-aware video understanding depends on the diffusion of heterogeneous multimedia data, where an artificially intelligent algorithm is employed to recognize the concepts from the video message. This results in overall precise concepts, emotions, and temporal and categorical understanding of the video. The generated concepts of the context-aware video understanding technique are precise, determined, and clear compared to the general concepts obtained from the deep learning technique. These improvements are accomplished by utilizing less time, computing power, and data. This makes our approach fit better in real-time scenarios, where a fast decision needs to be made. Besides this subjective comparison, an objective comparison is presented to show clearly how our proposed technique outperforms the learning-based algorithm.

Future work shall consider merging relevance feedback in the video understanding process. Relevance feedback is a practical and applicable way to represent human preferences because the relevancy of a video file is a user's opinion. In that case, similarity measures could be used to assess the relevancy of the video file content. A heterogeneous artificial intelligent system composed of context-awareness, deep learning, relevance feedback, and similarity measures would enable a human-like intelligent performance.

## Figures and Tables

**Algorithm 1:**
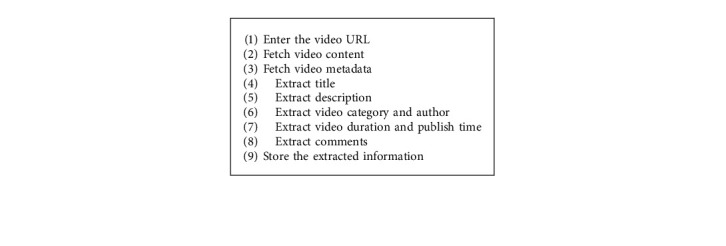
Retrieve video context metadata.

**Algorithm 2:**
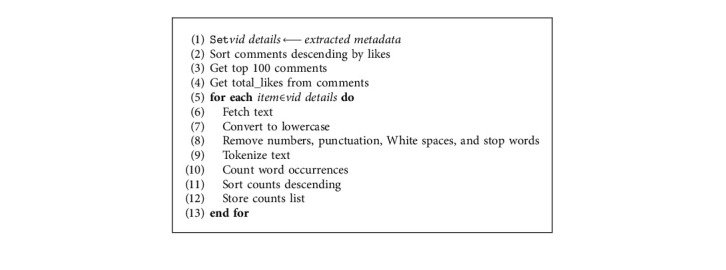
Process video context metadata.

**Algorithm 3:**
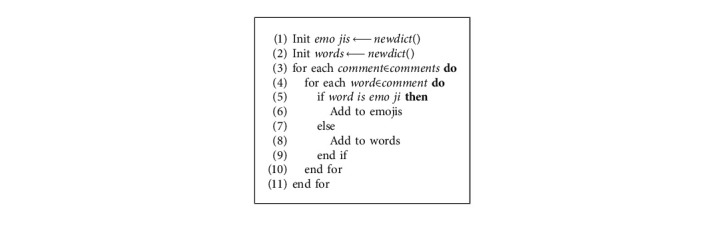
Segregate words and emojis.

**Algorithm 4:**
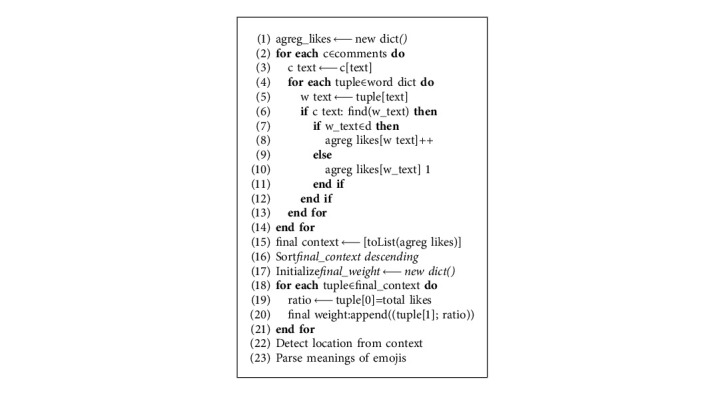
Generate concepts from context.

**Table 1:** Screaming kid dataset collected from YouTube.

Video ID	Title	YouTube ID	Duration	Author
1	How To Get Your Kid To Stop Screaming	kc7YmtLLlu0	10:45	Live On Purpose TV
2	Psycho Kid Ruins thanksgiving	TUCUsNx1HTs	03:40	McJuggerNuggets
3	Teen screams during bond hearing	uzKT50sYHtQ	02:23	WKMG News 6 ClickOrlando
4	The biggest Rage ever in GMOD! (Garry's Mod Trolling)	QJHfqPC-L20	04:32	SgtSizzles
5	Kid Screaming Tantrum (G MAJOR + EAR RAPE)	NAjw31iNpPw	00:39	G Major Manager
6	TEEN SHOUTS AT ANNOYING BRAT	vAf6J9qstN4	04:37	The Shame Game
7	Funny kid screaming	LkpI8RNUeYg	00:38	Harry Mcclean
8	Sonic Kid Screaming	FOYZvSXpja0	00:05	Haise Sasaki
9	Just noticed bts put screaming boy in “Not Today”	hCMSGRrhk8k	00:48	EpicGH
10	Water Wake Up Prank Makes Kid Scream Like a Banshee!	L5Tq8MCT9OU	00:39	PRNK

Video duration is in minutes. Full URL to a video is obtained by replacing the ID portion in YouTube base URL: https://www.youtube.com/watch?v=<ID>.

**Table 2:** Concepts extracted from the context.

Video ID	Concepts extracted from the context	
	Top 5 concepts	Less weighted concepts
1	0.197–>video, 0.197–>parent, 0.105–> need, 0.076-> watch, 0.057–>learn	Take, stop, scream, child, help
2	0.311–>video, 0.278–>like, 0.198–>thanksgiving, 0.104-> psycho, 0.030 –>dinner	Want, ruin, today, family, seri
3	0.710–>scream, 0.137–>hear, 0.049 –>judge, 0.036-> murder, 0.036–>bond	Roommate, teen
4	0.801–>ever, 0.192–>gmod, 0.004–>rage, 0.002–>subscribe	None
5	0.5–>major, 0.25-> watch, 0.25–>video	None
6	0.081–>child, 0.06-> flight, 0.060–>attend, 0.057–>other, 0.056–>like	Play, annoy, talk, brat, shut
7	None	None
8	None	None
9	0.946–>scream, 0.036–>video, 0.0178–>today	None
10	0.376–>water, 0.261–>scream, 0.138-> like, 0.130–>banshe, 0.029–>sleep	Wake, funny, make, wrong, reaction

**Table 3:** Emotional, categorical, temporal, and spatial information about the video file. These specifications are extracted from the context and by no means can be extracted by deep learning.

Video ID	Emotions	Temporal	Spatial	Category
1	Joy, laughing, thumbs up, proud	2018-01-31 17:01:13	None	Education
2	Joy, laughing	2014-11-27 20:00:14	None	Comedy
3	Insane	2018-12-10 11:51:52	None	News & politics
4	Joy, laughing	2016-04-24 14:25:27	None	Gaming
5	None	2018-10-07 18:23:34	None	People & blogs
6	Joy, laughing, smiling	2019-08-15 09:41:52	None	Entertainment
7	None	2013-02-22 20:40:23	None	People & blogs
8	None	2019-04-13 00:04:20	None	People & blogs
9	Joy	2018-01-09 19:50:25	None	Film & animation
10	Joy, laughing, sweet smile	2015-06-14 17:00:01	None	People & blogs

**Table 4:** Actions recognized by deep learning versus concepts extracted from the context.

Video ID	Concepts extracted from the context	Actions recognized by deep learning
1	0.197 –>video, 0.197 –>parent, 0.105 –>need, 0.076 –>watch, 0.057 –>learn	0.302 –>lecturing, 0.112 –>adult + male + speaking, 0.069 –>pointing, 0.041 –>teaching, 0.036 –>discussing
2	0.311 –> video, 0.278 –>like, 0.198 –>thanksgiving, 0.104 –>psycho, 0.030 –>dinner	0.078 –>dining, 0.051 –>drinking, 0.044 –>discussing, 0.044 –>serving, 0.031 –>autographing
3	0.710 –>scream, 0.137 –>hear, 0.049 –>judge, 0.036 –>murder, 0.036 –>bond	0.135 –>discussing, 0.113 –>pointing, 0.075 –>arresting, 0.065 –>adult + male + speaking, 0.035 –>talking
4	0.801 –>ever, 0.192 –>gmod, 0.004 –>rage, 0.002 –>subscribe	0.202 –>aiming, 0.111 –>mowing, 0.046 –>playing + videogames, 0.040 –>loading, 0.040 –>pointing
5	0.5 –>major, 0.25 –>watch, 0.25 –>video	0.132 –>shopping, 0.041 –>stealing, 0.029 –>buying, 0.027 –>playing, 0.023 –>child + speaking
6	0.081 –>child, 0.06 –>flight, 0.060 -–>attend, 0.057 –>other, 0.056 –>like	0.097 –>waxing, 0.059 –>taping, 0.035 –>cleaning, 0.028 –>pressing, 0.027 –>preaching
7	None	0.126 –>bicycling, 0.062 –>juggling, 0.038 –>running, 0.037 –>feeding, 0.036 –>kicking
8	None	0.053 –>dancing, 0.050 –>adult + male + singing, 0.049 –>playing + videogames, 0.044 –>wrestling, 0.035 –>adult + female + singing
9	0.946 –>scream, 0.036 –>video, 0.0178 –>today	0.061 –>adult + male + singing, 0.047 –>twisting, 0.040 –>racing, 0.031 –>driving, 0.027 –>performing
10	0.376 –>water, 0.261 - > scream, 0.138 –>like, 0.130 –>banshe, 0.029 –>sleep	0.093 –>tickling, 0.053 –>laughing, 0.026 –>giggling, 0.026 –>adult + male + speaking, 0.024 –>juggling

**Table 5:** Time consumed for retrieving video context metadata versus video content, measured in seconds.

Video ID	Video metadata	Video content
1	6.422449	43
2	10.155952	45
3	16.433475	21
4	8.415379	126
5	5.817539	42
6	6.704641	47
7	6.02976	18
8	6.646857	9
9	5.895031	17
10	5.612696	15

**Table 6:** Processing time for video context metadata versus video content, measured in seconds.

Video ID	Video context metadata	Video content
1	4.422285	79
2	1.127109	34
3	1.125429	27
4	0.929536	57
5	0.79569	15
6	5.05579	71
7	0.401577	11
8	0.006824	10
9	0.777581	16
10	1.362068	16

**Table 7:** Processed data size in deep learning and context-awareness.

Video ID	Metadata (KB)	Content (MB)
1	391.5	91.2
2	294	89.1
3	275	34.1
4	257	166
5	204.9	20
6	230	90.2
7	926	18
8	155	1.8
9	188.5	9.3
10	28	11

## Data Availability

The data used to support the findings of this study are available inside the paper.
